# Heart Rate and its Variability Assessed by Spectral Analysis in Elderly Subjects with Orthostatic Hypotension: A Case-Control Study

**DOI:** 10.5935/abc.20180043

**Published:** 2018-04

**Authors:** Rose Mary Ferreira Lisboa da Silva, Carlos Eduardo de Souza Miranda, Maira Tonidandel Barbosa, Maria Aparecida Camargos Bicalho

**Affiliations:** Faculdade de Medicina da Universidade Federal de Minas Gerais, Belo Horizonte, MG - Brazil

**Keywords:** Heart Rate, Hypotension, Orthostatic, Accidental Falls, Syncope, Aged, Dizziness

## Abstract

**Background:**

The prevalence of orthostatic hypotension (OH) increases with age and is associated with changes in autonomic regulation of blood pressure (BP) and heart rate (HR).

**Objective:**

to assess HR and HR variability (HRV) in elderly subjects with OH and determine OH predictors.

**Methods:**

a total of 105 patients aged ≥ 60 years, 39 with OH (case group) and 66 without OH (control group) (age-matched) were studied. Patients underwent clinical assessment, electrocardiogram, biochemistry tests and Holter monitoring for spectral analysis of HRV (Fourier transform) in the supine and orthostatism positions to identify low frequency (LF) and high frequency (HF) components, as well as the LF/HF ratio.

**Results:**

median age was 73.0 years, 64 patients were women. In all participants, there was a reduction in HF (133.0 versus 76.0 ms^2^, p = 0.001) and increase in LF/HF (1.6 vs 2.1; p < 0.001) and no change in LF (233.0 versus 218.0 ms^2^, p = 0.080). Between-group comparisons revealed significant differences in the median values of HR in the supine position (62.0 vs. 69.0 bpm, p = 0.001) and LF in the supine position (157.0 in case group vs. 275.0 ms^2^ in the control group, p = 0.014). Spearman’s correlation coefficient of 0.27 was found between the groups. Multivariate analysis revealed that HR in the supine position was an independent variable for OH (p = 0.001- 95%CI = -0.022 and -0.006). Using the operating characteristic curve, the best cutoff point was 61 bpm, with a sensitivity of 77.3% and specificity of 51.3%, positive predictive value of 61.3%, and negative predictive value 69.3%. Odds ratio was 3.23 for OH in patients with a HR lower than 61 bpm.

**Conclusions:**

lower LF and HR in the supine position were found in patients with OH, regardless of age and gender. The independent predictor for OH was HR in the supine position, with an odds ratio of 3.23 for values lower than 61 bpm.

## Introduction

Orthostatic hypotension (OH), also known as postural hypotension is defined as a sustained fall in blood pressure (of at least 20 mm Hg in systolic pressure and/or at least 10 mm Hg in diastolic pressure) occurring within 3 minutes of standing.^1,2^ OH has been associated with falls, presyncope, syncope, functional impairment in the elderly, cardiovascular events, and increased mortality.^3-5^ Its prevalence varies from 6 to 35%,^4^ and can achieve 41% in individuals aged 80 years or older.^6^

With aging, there are changes in autonomic regulation of heart rate (HR) and blood pressure. Middle-aged women have a more dominant parasympathetic whereas men have a more sympathetic regulation of heart rate.^7^ In addition, increased levels of norepinephrine and reduced sensitivity of beta-adrenergic receptors are found in elderly subjects. There is a decrease in vasomotor response mediated by alpha receptors, with decline in venous capacitance response of the lower limbs and in baroreflex response, which is also due to artery stiffness.^8,9^ This altered regulation lead to autonomic dysfunction and may cause OH. Autonomic nervous system, involved in the physiopathology of OH, may be examined by measurements of the heart rate variability (HRV) by Holter monitoring,^10^ a non-invasive, low-cost method.

Studies on the autonomic nervous system, including those on baroreflex sensitivity and head-up tilt test have been performed in hypertensive and normotensive elderly patients who were compared with young subjects. These studies included up to 80 elderly subjects, 64 with hypertension.^11-13^ In the largest study, involving 362 volunteers, there were 38 men and 51 women aged between 57 and 88 years,^13^ but the authors did not specify the exact number of older patients. In light of this, this study aimed to assess HR and HRV by spectral analysis during postural tilt in elderly patients, and evaluate OH predictors.

## Methods

This was an observational, prospective, cross-sectional study. Our sample was composed of 105 outpatients aged 60 years or older, included during the period from February 2013 to August 2014. Patients with dementia, autonomic dysfunction-related neurologic diseases, persistent or permanent atrial fibrillation, pacemakers, institutionalized patients, and those using antiarrhythmic agents (Class I, III or IV agents according to Vaughan Williams classification) or digoxin were excluded.

The study was approved by the local Research Ethics Committee and all participants signed the informed consent form.

For sample estimation, a one-tailed test was used, with significance level at 5%, power of 90%, two controls per case, frequency of OH of 30%. Two groups, age matched, were studied - a case group (n = 39) with OH, and a control group (n = 66) without OH.

Participants underwent clinical assessment, clinical pathology tests, 12-lead electrocardiography, measurements of blood pressure (BP) at supine position at the 5^th^ minute of rest and at the 3^rd^ minute of orthostatism or before, in case they had OH symptoms according to well-established conditions,^2^ monitored by the Holter system. Measurements were performed at a temperature-controlled room, in the afternoon, at least two hours after lunch to exclude the possibility of post-prandial hypotension. Holter monitoring was performed using a three-channel digital recorder (Cardioflash) (modified V1 and V5 and DIII) version 1.0, at supine and orthostatic positions for 15 and 10 minutes, respectively, for analysis of HRV in the frequency domain by the fast Fourier transform method. Measurements of high-frequency (HF) and low-frequency (LF) components that indicate parasympathetic and sympathetic activities, respectively, as well as the LF/HF ratio^10^ were calculated. This analysis was performed after manual edition of recordings to remove artifacts and correct arrhythmias. Measures were obtained during 5 minutes at 10^th^ minute of supine position and 5^th^ minute of orthostatic position. Results of the spectral analysis were expressed in ms^2^.

The Framingham^14^ and the PROCAM^15^ risk scores were also calculated using clinical and laboratory data, which included plasma levels of cholesterol and its fractions, triglycerides, and fasting glucose levels.

### Statistical analysis

For data analysis, we used the International Business Machines (IBM) Statistical Package for Social Sciences (SPSS) Statistics 19. Results were expressed as numbers and proportions for categorical variables and as central tendency (mean and median) and dispersion measures for continuous variables. Associations between categorical variables were assessed by the chi-square test or the Fisher’s exact test, as appropriate. Data normality was not tested. The Mann-Whitney test was used for comparisons between continuous variables, and correlations between categorical variables were assessed by the Spearman's rank correlation test. The Wilcoxon test was used to compare the two periods HRV components in the spectral analysis (supine and orthostatic positions). Stepwise multivariate analysis was performed to evaluate predicting values of OH, considering the variables with a p ≤ 0.10 in the univariate analysis. Receiver operating characteristic curve was analyzed for the stable variable postural response. The level of significance was set at 5%.

## Results

### General characteristics of the population

Mean and median age were 71.9 and 73.0 years, respectively; 64 (61%) were women. Clinical variables of the study population are described in Table 1.

**Table 1:** Anthropometric and clinical variables of studied patients

Variables	Median	Interquartile range Q1 - Q3	Minimum value	Maximum value
Age (years)	73.0	65.5 - 77.0	60.0	91.0
Weight (kg)	62.0	56.0 - 72.0	44.0	102.0
Height (m)	1.58	1.51 - 1.62	1.41	1.80
BMI (kg/m^2^)	25.7	22.5 - 29.7	17.8	40.9
WC (cm)	87.3	80.3 - 96.0	68	116
HR supine position (bpm)	68.0	60.0 - 76.0	38.0	105.0
HR orthostatic (bpm)	72.0	64.0 - 80.0	44.0	109.0
SAP supine position (mmHg)	140.0	127.0 - 152.0	92.0	196.0
DAP supine position (mmHg)	80.0	75.0 - 87.0	60.0	104.0
SAP orthostatic position (mmHg)	130.0	120.0 - 142.0	60.0	220.0
DAP orthostatic position (mmHg)	80.0	70.0 - 90.0	30.0	100.0
SAP seated position (mmHg)	135.0	120.0 - 150.0	100.0	194.0
DAP seated position (mmHg)	80.0	70.0 - 90.0	60.0	106.0

BMI: body mass index; WC: waist circumference; HR: heart rate; bpm: beats per minute; SAP: systolic arterial pressure; DAP: diastolic arterial pressure; mmHg: millimeter of mercury; Q1: 25^th^ percentile; Q3: 75^th^ percentile

With respect to cardiovascular risk factors, systemic arterial hypertension (SAH) and dyslipidemia were the most frequent, found in 80 (76.2%) and 42 (40%) patients, respectively. Diabetes was found in 17.1% of patients.

Thiazide diuretics were the most used antihypertensive drugs; 42 patients (40%) used them isolated or in combination with other antihypertensive agents. Following thiazide diuretics, angiotensin II receptor blockers (29.8%), angiotensin-converting enzyme (ACE inhibitor) (28.6%) and beta-blockers (27.6%) were the most common, with similar frequencies of use. Also, 14.3% of patients were using calcium antagonists (amlodipine or nifedipine).

Symptoms characterized by previous history of dizziness, falls, and presyncope and/or syncope were reported by 64 patients (61%).

Impaired conduction in the left bundle branch was detected at electrocardiography in 9.5% of patients, with mean PR and QT intervals of 166.9 ms (120-280) and 403.0 ms (320-520), respectively.

### Comparison between case and control groups

No difference was found in age (mean of 73.5 ± 8.0 years; median of 74.0 in the case group and 71.0 ± 6.8 years and 72.0 years in the control group, p = 0.119), but a significant difference in sex was observed between the groups (56.4% of men in the case group and 27.8% of men in the control group, p = 0.005). No correlation was found between these two variables (Spearman’s coefficient correlation of 0.274).

Results of other comparisons between the two groups are described in Table 2. No patient had dizziness, presyncope or syncope in orthostatism when BP was measured. No difference considered abnormal in BP between the upper limbs was detected in seated position.

**Table 2:** Between-group comparison of heart rate, blood pressure and cardiovascular risk scores

Variables	Case group Median (Q1 - Q3)	Control group Median (Q1 - Q3)	p-value
HR supine position (bpm)	62.0 (57.0 - 72.0)	69.0 (63.5 - 76.0)	0.001
HR orthostatism (bpm)	67.0 (60.0 - 76.0)	75.0 (68.0 - 80.0)	0.006
SAP supine position (mmHg)	140.0 (130.0 - 160.0)	135.0 (125.8 - 150.0)	0.189
DAP supine position (mmHg)	80.0 (70.0 - 90.00	80.0 (78.0 - 86.0)	0.543
SAP orthostatism (mmHg)	120.0 (110.0 - 135.0)	136.5 (120.0 - 146.2)	0.001
DAP orthostatism (mmHg)	72.0 (60.0 - 84.0)	80.0 (77.3 - 90.0)	0.001
Framingham score	15.5 (6.0 - 24.3)	12.0 (6.0 - 17.0)	0.063
PROCAM score	10.6 (5.01 - 21.4)	11.0 (5.0 - 16.8)	0.537

SD: standard deviation; HR: heart rate; bpm: beats per minute; SAP: systolic arterial pressure; DAP: diastolic arterial pressure; mmHg: millimeter of mercury. Mann‑Whitney test; Q1: 25^th^ percentile; Q3: 75^th^ percentile

Significant differences were found in the frequency of previous symptoms (dizziness, prepsyncope and syncope) - 77% in the case group *versus* 51.5% in the control group (p < 0.001). However, no difference between patients with and without previous symptoms were found in age (mean or median) - 71.4 ± 7.4 years; 72.0 years *versus* 72.7 ± 7.8 years, 74.0 years; respectively (p = 0.38) - nor in BP measured in the supine position.

With respect to hypertension, no difference was found between the case and control groups (p = 0.54). Forty-nine patients (74.2%) in the control group and 31 in the case group were hypertensive (79.4%). There was no difference in the frequency of diabetes (7 patients in the case group and 11 in the control group; p = 0.86) or coronary arterial disease (5% in the case group and 9% in the control group) between the groups. All patients were stable, without chest pain.

Regarding the main groups of antihypertensives, higher percentage of users of ACE inhibitors was observed in the case group (41.0%) than in the control group (21.2%) (p = 0.030). No difference was found in other antihypertensive agents.

### Heart rate variability

Medians and interquartile ranges of HRV components in supine position were - LF 233.0 ms^2^ (130.5 - 422.5), HF 133.0 ms^2^ (62.0 - 347.5), LF/HF 1.6 (0.8 - 3.0) - and in orthostatic position were - LF 218.0 ms^2^ (110.5 - 359.7), HF 76.0 ms^2^ (32.0 - 227.0) and LF/HF 2.1 (1.1 e 4.8). Comparisons of HRV components between supine and orthostatic positions performed by the Wilcoxon test showed no difference in LF (p = 0.080), but significant differences in HF (p = 0.01) and LF/HF (p < 0.001).

When HRV absolute values were compared between case and control groups by the Mann-Whitney test, significant difference was found in LF in supine position (Table 3). No difference between the groups was found in other components. Due to HRV data interval, a logarithmic transformation of HRV components was performed, and the same p-values were maintained. For analysis of HRV with change of position, median differences in LF component were compared between case and control groups (i.e. between the supine and the orthostatic position, median -0.27 ms^2^) by the Mann Whitney test (p = 0.43). Median differences of HF and LF/HF components were 33.0 ms^2^ and 0.53, respectively, and p-values of respective comparisons were 0.74 and 0.94.

**Table 3:** Comparison of heart rate spectral analysis between case and control groups

Variables	Case group Median (Q1 - Q3)	Control group Median (Q1 - Q3)	p-value
LF supine position (ms^2^)	157.0 (83.6 - 323.3)	275.0 (164.0 - 439.5)	0.014
HF supine position (ms^2^)	111.0 (50.5 - 368.5)	141.0 (65.0 - 342.5)	0.873
LF/HF supine position (ms^2^)	1.5 (0.7 - 2.4)	1.8 (0.9 - 4.1)	0.054
LF orthostatism (ms^2^)	161.5 (71.5 - 333.6)	242.0 (128.5 - 375.0)	0.075
HF orthostatism (ms^2^)	66.0 (29.0 - 229.5)	91.0 (33.5 - 247.1)	0.898
LF/HF orthostatism (ms^2^)	1.8 (1.0 - 3.3)	2.4 (1.2 - 6.1)	0.096

SD: standard deviation; LH: low frequency; HF: high frequency; LH/HF: low frequency/high frequency ratio; ms: milliseconds. Mann-Whitney test; Q1: 25^th^ percentile; Q3: 75^th^ percentile

**Figure 1:**
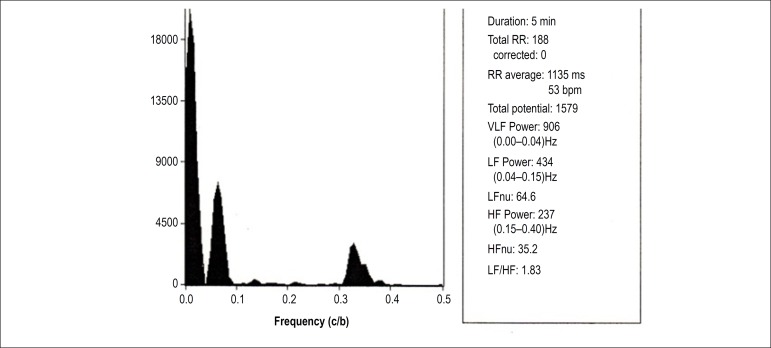
Spectral analysis of a male patient (67 years of age) with orthostatic hypotension in supine position. RR: number of QRS in sinus rhythm; VLF: very low frequency; LF: low frequency; HF: high frequency; HFnu: HF normalized unit.

**Figure 2:**
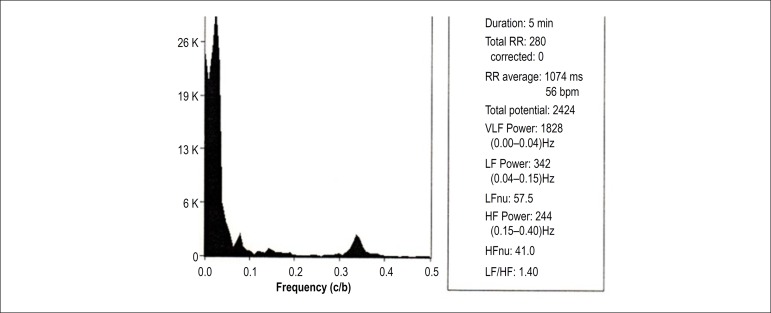
Spectral analysis of a male patient (67 years of age) with orthostatic hypotension (same of Figure 1) in orthostatic position, showing a decrease in low frequency (LF) component and in the LF/high frequency (HF) ratio, in relation to supine position. RR: number of QRS in sinus rhythm; VLF: very low frequency; HFnu: HF normalized unit.

**Figure 3:**
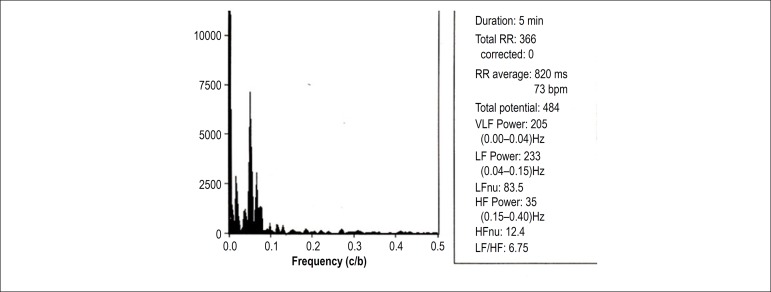
Spectral analysis of a female patient (62 years of age) without orthostatic hypotension in supine position. RR: number of QRS in sinus rhythm; VLF: very low frequency; LF: low frequency; HF: high frequency; HFnu: HF normalized unit.

**Figure 4:**
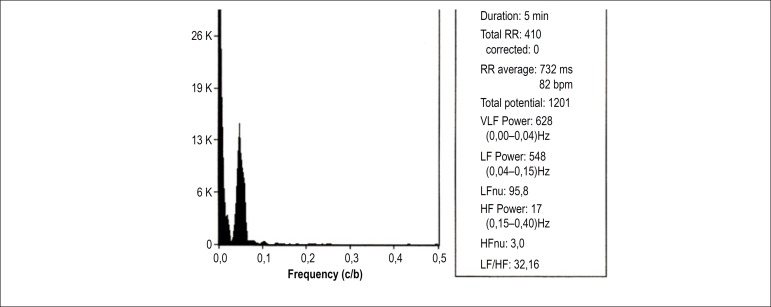
Spectral analysis of a female patient (62 years of age) without orthostatic hypotension (the same of Figure 3) in orthostatic position, showing an increase in low frequency (LF) component and in the LF/high frequency (HF) ratio, in relation to supine position. VLF: very low frequency; RR: number of QRS in sinus rhythm; VLF: very low frequency; HFnu: HF normalized unit.

Figures 1 and 2 depict the analysis of HRV components of a patient with OH in the supine and orthostatic positions, respectively. Figures 3 and 4 show the analysis of HRV components of a patient without OH in the supine and orthostatic positions, respectively.

### Stepwise multivariate analysis

Variables with a p ≤ 0.10 in the univariate analysis - sex, use of ACE inhibitors, presence of previous symptoms, HR, LF and LF/HF in supine and orthostatic positions, and Framingham score - were considered for the multivariate analysis. The independent variable with statistical significance was HR in the supine position, with p = 0.001, 95% confidence interval of -0.0022 - -0.006.

### Analysis of the receiver operating characteristic curve

**Figure 5:**
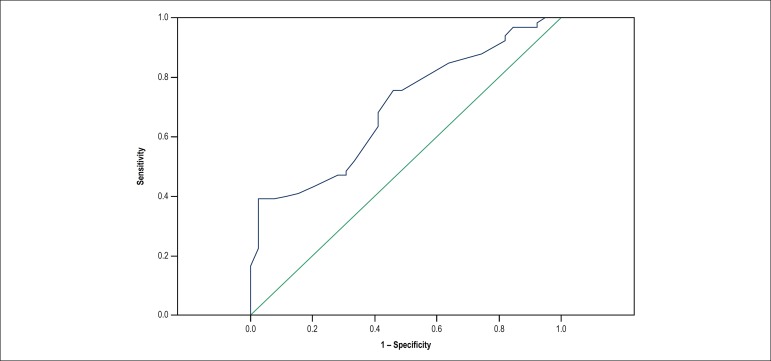
Receiver operating characteristic curve of heart rate in the supine position (blue line), considering the stable variable postural response without orthostatic hypotension.

Using the receiver operating characteristic curve for the stable variable postural response without OH, and considering the variable HR in the supine position, an area under the curve of 0.70 was obtained (Figure 5), with a p = 0.001 (95% confidence interval of 0.595-0.796). The best cutoff point was 61 bpm, with a sensitivity of 77.3% and specificity of 51.3%. Positive predictive value was 61.3%, and negative predictive value 69.3%. Odds ratio was 3.23 for OH in patients with a HR lower than 61 bpm.

## Discussion

The main finding of this study was that, in contrast to HRV components, HR in the supine position was an independent predictor for the occurrence of OH in the study population. Median HR in the supine position was significantly lower in the case group than in the control group in the same position. Although this variable was a predictor of OH, with an odds ratio of 3.23 for patients with HR < 61 bpm, it was not considered a good diagnostic test, as confirmed by the analysis of the receiver operating characteristic curve.

Aging is one of the main predicting factors for OH, which may be explained by changes in the autonomic regulation of BP and HR. Increases in norepinephrine plasma levels, decreased sensitivity of beta-adrenergic receptors, decreased vasomotor response mediated by alpha receptors, and reduced baroreflex response and parasympathetic tone contribute to the occurrence of OH in the elderly.^8,9^ Therefore, while approximately 5% of middle-aged adults have OH,^16^ the prevalence increases to nearly 41% in those aged 80 years or older.^6^ In the present study, patients with and without OH were age-matched and all of them were younger than 60 years, and hence results were not affected by age.

Previous studies have shown different results in the prevalence of OH and its association with gender, according to age and study setting. In a study conducted in the 90’s decade, Rutan et al.^17^ evaluated a population of 5,201 elderly subjects (≥65 years), with an OH prevalence of 18.2% and no difference between the sexes. In patients hospitalized for OH, the prevalence was higher in men (55.3% in the age range of 65 - 74 years), although 54% of patients aged 75 years or older were women, with a total of 15,858 admissions for OH in a year.^18^ In our population, there was a predominance of men in the group of patients with OH; however, Spearman’s correlation coefficient was 0.27, i.e., of small magnitude, which suggest that there was no statistical difference in sex between the groups. Also, sex was not an OH predictor in multivariate analysis.

Studies have demonstrated the importance of rises in HR in the orthostatic position, its association with increased sympathetic activity and higher orthostatic tolerance.^19^ Nevertheless, the role of HR in the supine position in OH patients has not been well established yet. Changes in autonomic tone^13^ and sinus node dysfunction^20^ may be associated with reduced HR in supine position in elderly patients, regardless of OH. In the study population, when this variable was analyzed, median HR in supine position in the case group was significantly lower than in control group. In addition to the changes already described, another factor that may be associated with differences between the groups would be the use of beta-blockers. These medications have a negative chronotropic effect on HR.^21^ However, in the present study, no difference was found in the use of most of these drugs between the groups. Significant difference was found in the use of ACE inhibitors, which decrease vascular resistance but have no substantial effect on HR, despite restoration of parasympathetic tone with the use of the drug in hypertensive patients.^22^

In light of this, analysis of HRV was important for the study of HR profile in elderly subjects with OH in supine position. With change of position, as expected,^10^ there was a decrease in HF and increase in LF/HF in all patients, without an increase in the LF component. There is a decline in baroreflex and HRV with age in both sexes, resulting in autonomic dysfunction.^6,8,13^ A U-curve has been used to describe the progressive decrease of HRV with aging, which reaches its nadir in the sixth or seventh decade of life, followed by progressive increase, which is determinant for longevity from those decades on.^23-26^ Therefore, to avoid age bias, the case and control groups were matched by age. Although previous studies on OH and HRV^11-13,27^ have shown different results, it is possible to infer from their results that autonomic system and baroreflex dysfunction are associated with OH. Harrington et al.^11^ evaluated baroreflex sensitivity by digital plethysmography in the elderly - 75 normotensive and 64 hypertensive patients, without medications. The authors reported reduced baroreflex, with impairment of HF component. Kawaguchi et al.^12^ shown a decrease in the LF/HF ratio and decreased cerebral perfusion measured by infrared spectroscopy in a group of 80 elderly subjects after passive standing. A more recent study on hypertensive patients,^27^ 18 with OH and 64 without OH (mean age of 74.2 years), demonstrated that, despite lower systolic volume in those with OH, no significant differences in change in the LF/HF ratio after orthostatism were found, which is in agreement with our results. Barantke et al.^13^ demonstrated a decline in all components of HRV with age, and an association between LF and baroreflex sensitivity in orthostatic position. This evidence that LF reflects baroreflex function rather than sympathetic innervation measured by 6-[18F] fluorodopamine has also been demonstrated by other authors.^28,29^ Consequently, our findings suggest the hypothesis that the lack of increase in HR and LF with orthostatism may be related to baroreflex dysfuntion,^27,30^ which predisposes to OH.

Blood pressure may also influence the prevalence of OH. Studies on hypertensive elderly patients demonstrated higher prevalence of OH in those with higher BP levels. Gangavati et al.^31^ followed 722 elderly patients and found a prevalence of OH of 19% in participants with uncontrolled hypertension (BP ≥ 140/90 mmHg) and of 5% in those with controlled hypertension (BP < 140/90 mmHg). Mean age was 78 years in both groups. Valbusa et al.^32^ reported similar findings but with different OH prevalence - the authors evaluated 994 patients with mean age of 88 years; the prevalence of OH was 13% in hypertensive patients with BP ≤ 140/90 mmHg and 23% in those with BP > 140 mmHg. In the present study, no difference between the groups was found in baseline BP in the supine position or in the prevalence of hypertension.

Regarding the association of OH with the use of medications, in a study with 189 patients aged 75 or older with OH, the prevalence of OH was of 35%, 58%, 60% and 65% in those patients using none, one, two, three or more medications, respectively. Although the study included medications other than anti-hypertensive agents, hydrochlorothiazide was associated with higher prevalence of OH (65%).^33^ Analysis of a cohort of 3,775 women aged between 60 and 80 years demonstrated that the use of three or more anti-hypertensive agents had a 2.2 greater chance of developing OH in comparison with patients taking no medications.^34^ In the present study, although the use of ACE inhibitors was significantly higher in patients with OH, this drug was not a predictor of this condition, which may be explained by its role on autonomic modulation.^22^

Diabetes mellitus may also result in autonomic dysfunction.^4^ In our study, its prevalence was 17.1% in the population, with no difference between the groups and, thereby, had no influence on the results.

As previously reported, clinical manifestations of OH that may lead to falls, fractures, presyncope and syncope cause functional impairment in the elderly, which is known as frailty syndrome.^3-5^ In the current study, previous symptoms including dizziness, presyncope and syncope were more frequent in patients in the case group and, according to the literature, these symptoms may be associated with frailty syndrome and lower BP values after orthostatism.^4,35^

Data in the literature on the association between frailty and risk for cardiovascular disease are scarce. A study on 1,622 elderly men aged between 71 and 92 years showed an association between frailty and increased risk factors, including waist circumference, lipid profile and SAH, despite similar prevalence of these factors between frail and non-frail elderly persons. Cardiovascular risk scores were not calculated, but this association was independent of established cardiovascular disease.^36^ In the present study, patients were assessed for cardiovascular risk using the Framingham^14^ and PROCAM scores,^15^ with no difference between the groups. It is worth mentioning that 75 years is the age limit for the use of these scores.

### Limitations

The main limitations of this study were the number of patients and the fact that they were assessed only once, which made the evaluation of reproducibility of results impossible. The use of digital plethysmography for measurement of BP levels in orthostatic position would enable the early detection of OH. Besides, we did not evaluate the very low frequency (VLF) component of HRV, associated with renin-angiotensin-aldosterone system, thermoregulation and peripheral vasomotor tone.

## Conclusions

In the study population, lower LF and HR in the supine position was found in patients with OH, regardless of gender, BP in supine position and use of beta-blockers. HR in the supine position was an independent predictor for OH with an odds ratio of 3.23 for values lower than 61 bpm.
